# World Experience in Immunization against Noncommunicable Diseases: Successes and Vectors for Further Development

**DOI:** 10.3390/vaccines11081286

**Published:** 2023-07-27

**Authors:** Maria I. Kashutina, Inna A. Fadeeva, Yury V. Zhernov

**Affiliations:** 1 Department of Therapy, Clinical Pharmacology and Emergency Medicine, A.I. Yevdokimov Moscow State University of Medicine and Dentistry, 127473 Moscow, Russia; 2 Loginov Moscow Clinical Scientific and Practical Center, 111123 Moscow, Russia; 3 Department of Public Health Promotion, National Research Centre for Therapy and Preventive Medicine, 101990 Moscow, Russia; 4 Department of Foreign Language, Faculty of World Economy, Diplomatic Academy of the Russian Foreign Ministry, 119034 Moscow, Russia; 5 Department of Public Administration in Foreign Policy, Diplomatic Academy of the Russian Foreign Ministry, 119034 Moscow, Russia; 6 Department of General Hygiene, F. Erismann Institute of Public Health, I.M. Sechenov First Moscow State Medical University (Sechenov University), 119435 Moscow, Russia; 7 Department of Chemistry, M.V. Lomonosov Moscow State University, 119991 Moscow, Russia; 8 Center of Life Sciences, Skolkovo Institute of Science and Technology, 121205 Moscow, Russia; 9 Center for Medical Anthropology, N.N. Miklukho-Maclay Institute of Ethnology and Anthropology, Russian Academy of Sciences, 119017 Moscow, Russia; 10 Department of Medical and Biological Disciplines, Reaviz Medical University, 107564 Moscow, Russia

According to the World Health Organization, noncommunicable diseases (NCDs), also known as chronic diseases that do not spread from person to person, are one of the major burdens on public health and cause approximately 28 premature deaths worldwide every minute and close to 74% of deaths globally each year [1,2]. Due to the worse prognosis for the course of COVID-19 and the risk of developing long-term COVID in individuals with a history of NCDs, the COVID-19 pandemic has once again demonstrated the significance of NCDs in the structure of morbidity and mortality, confirming the need for timely disease detection and treatment [3]. The modern lifestyle brought on by the processes of urbanization, industrialization, and automation, characterized first and foremost by the presence of modifiable behavioral (e.g., sedentary lifestyle, unhealthy diet, lack of work/rest schedule, and predominance of daily distress), as well as metabolic risk factors (e.g., high blood pressure, overweight/obesity, hyperlipidemia, and hyperglycemia), contributes only to an increase in the prevalence and progression of the severity of NCDs [4,5]. According to Sustainable Development Goal 3.4, reducing premature mortality from NCDs by one-third by 2030 requires the implementation of new and improved prevention and treatment strategies. This is necessary to ensure sustainable socioeconomic development for all nations in the world [6].

Success in the fight against NCDs is challenging and requires the application of previously successful disease elimination approaches, including immunization. Currently, there are no vaccines available that can directly prevent NCDs such as cancer, heart disease, or diabetes. However, there are certain vaccines that can indirectly reduce the risk of developing these diseases by targeting specific factors associated with them. Human papillomavirus (HPV) vaccines have been proven to prevent infection with high-risk HPV types that can lead to cervical cancer and other HPV-related cancers such as anal, vaginal, vulvar, and oropharyngeal cancers [7]. Furthermore, the hepatitis B vaccine has been shown to reduce the risk of liver cancer [8], while the measles vaccine has been associated with a lower risk of asthma and allergies [9]. The influenza vaccine can indirectly reduce the risk of developing cardiovascular diseases [10], since influenza infection can trigger heart attacks and strokes. By preventing all these infections through vaccination, we can potentially reduce the burden of chronic diseases associated with them.

The development of vaccines specifically targeting noncommunicable diseases remains an area of ongoing research. Research is underway to develop vaccines targeting specific risk factors for heart disease, such as high cholesterol and high blood pressure. Cholesterol-Lowering Vaccination Strategies, such as active vaccines targeting Proprotein convertase subtilisin/kexin 9 (PCSK9) or apolipoprotein CIII (APOC3), have the potential to reduce the progression of atherosclerosis, the underlying cause of heart attacks and strokes, and prevent the development of cardiovascular events in at-risk individuals [11,12]. Active research is underway in the field of creating a vaccine against Alzheimer’s disease, where the target proteins are amyloid β and tau proteins [13]. A significant breakthrough has been made in the development of allergy vaccines based on recombinant allergens [14]. Some of them are now in the final stages of clinical research [15].

An important issue is the financial support for the process of prevention and treatment of NCDs, including immunization, which is a huge problem for the economy of any country. Thus, the estimated economic cost of the main NCDs is about 2 trillion US dollars per year, which exceeds the GDP of most G20 countries [16]. Immunization against NCDs is still a relatively new and evolving field. The development of vaccines for NCDs presents several challenges, including the identification of appropriate vaccine targets, understanding the immune response required for protection, determining the optimal vaccine dose and regimen, and ensuring the safety and efficacy of these vaccines. Additionally, there may be challenges in implementing immunization programs for NCDs, including issues related to vaccine acceptance, accessibility, and affordability. Despite all this, modern research shows that prevention strategies aimed at combating risk factors bring benefits in the long term and are relevant regardless of the region and country, and the cost-effectiveness of implementing integrated approaches to the prevention and control of NCDs is expected to be about 2.7 trillion dollars in 2023–2030 [17].

To fully harness the potential of immunization against NCDs, it is essential to invest in research and development, strengthen healthcare systems, and build public trust in vaccines. In conclusion, immunization against NCDs has the potential to be a game-changer in reducing the burden of these diseases globally.
